# Impacts of feeding milk replacer supplemented with increasing concentrations of choline on feed intake, growth, and scouring incidence for 7 weeks preweaning and 1 week postweaning

**DOI:** 10.3168/jdsc.2024-0557

**Published:** 2024-05-10

**Authors:** Anay D. Ravelo, Ellan I. Dufour, Makaila Klejeski, Bruce Ziegler, Angie Golombeski, Isaac J. Salfer

**Affiliations:** 1 Department of Veterinary Population Medicine, University of Minnesota, Saint Paul, MN, 55108; 2 Hubbard Feeds, Mankato, MN 56001; 3 Southern Research and Outreach Center, University of Minnesota, Waseca, MN 56093; 4 Department of Animal Science, University of Minnesota, Saint Paul, MN 55108

## Abstract

The objective of this study was to evaluate the effect of increasing choline supplementation on the intake of milk replacer (MR) and calf starter (CS), growth performance, and fecal scores of calves over 8 wk. One hundred calves from commercial herds were transported to the University of Minnesota Southern Research and Outreach Center (SROC) calf facility randomized to one of 4 treatments differing in choline supplementation within MR including: 0 (C0; n = 24), 700 (C700; n = 26), 1,400 (C1400; n = 25), and 2,100 mg/kg choline (C2100; n = 25). Milk replacer contained a basal concentration of 1,650 mg/kg MR resulting in total choline concentrations in the MR of 1,650 mg/kg for C0, 2,350 mg/kg for C700, 3,050 mg/kg for C1400, and 3,750 mg/kg for C2100. Calves were weaned at 49 d, and the trial ended at 56 d. Body weight was measured at d 0, 14, 28, 42, 49, and 56. Fecal scores were measured weekly. Feed intake was measured daily and calculated every 2 wk. All feeding, BW measurements, and fecal scoring were conducted by the research staff at the University of Minnesota SROC. Growth, feed intake, and gain to feed ratio were analyzed using a linear mixed effects model with choline concentration as a fixed effect and source herd and room as random effects. Initial BW was included as a covariate for average daily gain. Fecal scores were analyzed as a chi-squared test of significance. Feeding 700 mg/kg supplemental choline increased MR intake throughout the preweaning period and increased ADG in both the preweaning and postweaning periods compared with the other treatments. No differences were observed for fecal scores across treatments. Results suggest that calf performance can be improved with MR containing 2,350 mg/kg choline, contrasting with current NASEM (2021) requirements of 1,000 mg/kg.

## Highlights

• Supplementation of 700 mg/kg of choline to dairy calves increased average daily gain (ADG) and intake.
• Increasing choline supplementation did not affect scouring in calves.
• Supplementing above 700 mg/kg did not provide additional benefits to dairy calves.
• Total choline concentration in milk replacer was above the recommended 1,000 mg/kg DM.

## Graphical Abstract

**Figure:**
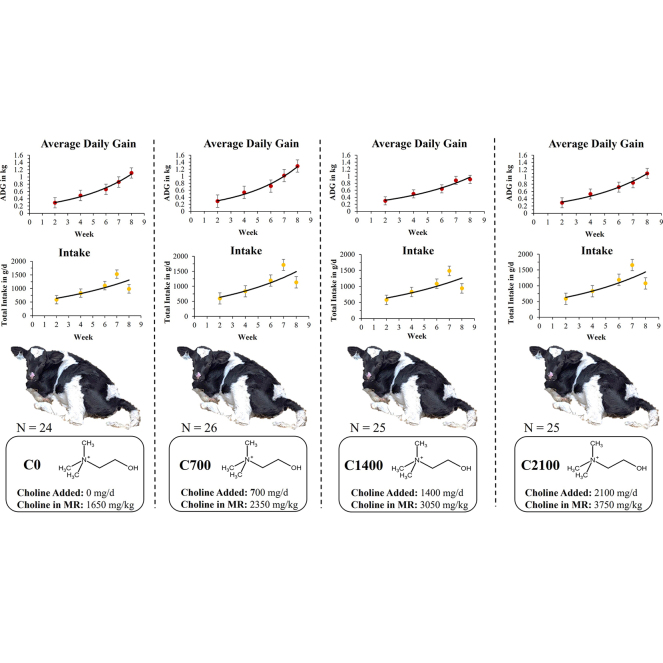
**Summary:** We investigated the effect of additional choline supplementation in dairy calves. There were 4 treatments: 0, 700, 1,400, or 2,100 mg/kg choline supplemented in milk replacer (MR). Average daily intake and MR intake were increased when choline was supplemented at 700 mg/kg, but not at greater amounts. All treatments had a basal choline concentration that was greater than the current National Academies of Sciences, Engineering, and Medicine (NASEM) recommendations of 1,000 mg/kg dry matter (DM). Although positive effects on growth were observed with 700 mg/kg supplementation, future research examining if similar effects could be accomplished with lower doses should be conducted to determine optimal choline requirements for calf growth and health.

Choline is a metabolically important quaternary ammonium cation that can be synthesized endogenously in the liver. However, in most animals, including dairy cows, hepatic synthesis is insufficient to meet requirements for the animal (Zeisel and Da Costa, 2009). Choline has multiple functions and supports production, reproduction, health, and growth in cattle. It is a major component of phospholipids and acetylcholine, which affects membrane integrity, and is involved in DNA methylation pathways (Pinotti et al., 2002; McFadden et al., 2020). Additionally, Abdelmegeid et al. (2017) observed in vitro that choline supplementation reduces pro-inflammatory activity and improves antioxidant balance in leukocytes from neonatal Holstein calves.

Although choline supplementation has been extensively researched in lactating dairy cows, specifically during the transition period, limited information on the role of choline supplementation in calves is available, especially in vivo. The current NASEM (2021) recommendation for choline requirements in calves is 1,000 mg/kg DM. To our knowledge, few studies have considered the impact of increasing choline supplementation on the intake of milk replacer (**MR**) and calf starter (**CS**), weight gain, and fecal scores of young preweaned calves. Thus, the objective of this study was to determine the effect of increasing choline supplementation on the intake of MR and CS, growth performance, and health of calves over 7 wk prepartum and 1 wk postpartum. We hypothesized that increased supplementation of choline would increase MR and CS intake, and BW gain, and reduce fecal scores of dairy calves.

To test this hypothesis, Holstein heifer calves (n = 100) aged at 2 to 5 d were sourced from 4 commercial dairy farms and transported to the University of Minnesota Southern Research and Outreach Center (**SROC**) Calf and Heifer Research Facility (Waseca, MN). Commercial farms were all located in southeast Minnesota within a 60-mile radius of SROC and were selected based on well-documented health records, the cleanliness of maternity facilities, and their compliance with appropriate colostrum feeding guidelines. Enrollment criteria required calves to be at least 48 h of age, have received 3 feedings of colostrum within 48 h of age, be free from apparent health issues, have a serum total protein concentration greater than 5, and have an initial BW between 34.0 and 47.6 kg. Calves were delivered to SROC twice per week, blocked by source herd, and randomly assigned to treatment upon arrival using Microsoft Excel (Microsoft Corp., Redmond, WA). All calves were enrolled on trial between July 12, 2022, and August 16, 2022, with the trial ending on October 14, 2022. Sample size was determined based on a >80% power of observing *P* < 0.05 difference in our primary outcome measure of ADG based on a standard deviation of 0.02 kg and an expected difference of 0.1 kg observed in previous experiments (Chester-Jones et al., 2016; Jaeger et al., 2020). Calves were housed in individual pens (2.3 × 1.2 m) with plastic side panels in one of 4 naturally ventilated nursery rooms with 2-curtain sidewalls. Calves were placed into nursery rooms sequentially by arrival date. All procedures for animal care and handling were approved by the University of Minnesota Institutional Animal Use and Care Committee (protocol number: 2110–39487A). Feeding, BW measurements, and fecal consistency scores were conducted by the previously trained research staff at the University of Minnesota SROC.

The same basal MR (Beginner Plus 24–20; Hubbard Feeds, Mankato, MN), containing (DM basis) CP: 24.8%, fat: 20.7%, NDF: 1.28%, ash: 7.91%, Ca: 1.22%, P: 0.75%, Mg: 0.15%, K: 1.56%, and S: 0.49%, while CS contained CP: 21.4%, starch: 35.2%, fat: 3.99%, NDF: 14.3%, ADF: 10.0%, ash: 6.55% (Wet Chemistry Analysis, Dairyland Laboratories Inc., Sauk Rapids, MN) was used for all treatments. Treatments included an all-milk protein MR supplemented with 0 mg/kg (**C0**; n = 24), 700 mg/kg (**C700**; n = 26), 1,400 mg/kg (**C1400**; n = 25), or 2,100 mg/kg (**C2100**; n = 25) choline. Basal MR contained 1,650 mg/kg choline, and total choline concentration in the MR for calves was 1,650 mg/kg DM for C0, 2,350 mg/kg DM for C700, 3,050 mg/kg DM for C1400, and 3,750 mg/kg DM for C2100. Choline was added directly to MR during production with final choline concentrations validated by the manufacturer. For each calf, d 1 of the trial started upon arrival at SROC and coincided with afternoon feeding. From d 1 to 7, 1.98 L or MR (12.5% wt/wt MR powder) was fed 2×/d at 0500 and 1500 h; from d 8 to 42, 2.38 L of MR (12.5% wt/wt MR powder) was fed 2×/d at 0500 and 1500 h; and from d 43 to 49, 2.38 L of MR (12.5% wt/wt MR powder) was fed 1×/d at 0500 h. Calves were weaned at 49 d. Refused MR was weighed daily. Calf starter (Elite 18% Texturized Calf Starter, Hubbard Feeds, Mankato, MN) containing decoquinate at 50.04 mg/kg was fed to maintain a 5% refusal rate beginning on d 1 of the study, with refusals determined daily. Calf starter contained no choline. Fresh water was offered twice daily throughout the duration of the study. Calves were removed from the trial on d 56 after arrival.

Upon arrival (d 1 of the trial), BW was measured using a digital scale (VS-660; A and A Scales LLC, Wyckoff, NJ), hip height was measured using a wooden measuring stick with sliding bar (Nasco Education, Fort Atkinson, WI), and 10 mL of blood was collected via jugular venipuncture into serum Vacutainer tubes (BD, Franklin Lakes, NJ), allowed to clot, centrifuged at 1,500 × *g* at 4°C for 15 min, and analyzed using a Brix refractometer (Spartan Refractometer, model A 300 CL, Spartan, Tokyo, Japan). Serum protein at arrival was 6.31 g/dL in C0, 6.11 g/dL in C700, 6.25 g/dL in C1400, and 6.38 g/dL in C2100 (*P* = 0.55). All calves with d 1 BW of less than 35.0 kg or greater than 47.6 kg were excluded from the study. Initial BW was 38.9 kg for C0, 40.3 kg for C700, 39.4 kg for C1400, and 39.7 kg for C2100 (*P* = 0.51).

Body weight was measured on d 14, 28, 42, 49, and 56 of the experiment at 1500 h. Gain to feed ratio (**G:F**) was calculated as kilograms of BW gain divided by kilograms of total feed intake. Hip height was measured on d 1 and 56, and hip height gain during the experiment was calculated. Daily fecal consistency scores were visually determined daily at 0500 h by SROC research staff using a 1 to 4 scale with 1 = normal, 2 = loose, pudding, 3 = very loose, no watery separation, and 4 = very loose, watery separation (Larson et al., 1977). Scouring frequency was determined as the number of days per week that the calves had a fecal score ≥3. Milk replacer intake, CS intake, total feed intake, ADG, G:F, and average fecal score were determined for each of the intervals between d 1 to 14, d 15 to 28, d 42 to 49, and d 49 to 56, for the preweaning period (d 1 to 49) and for the trial period (d 1 to 56). One calf was removed from treatment group C0 due to dramatic weight loss from d 1 to 7.

A linear mixed model was performed using the MIXED procedure of SAS 9.4 to determine effects of choline concentration on MR intake, CS intake, total intake, BW, ADG, G:F, hip height, and hip height gain during each of the weekly or every 2-wk time intervals. The model included the fixed effect of choline concentration and random effects of source herd and nursery room. Effects of choline concentration on daily MR intake, CS intake, total intake, ADG, and G:F over the preweaning period (d 1 to 49) and the entire trial period (d 1 to 56) were analyzed using repeated measures in the MIXED procedure of SAS with the fixed effect of choline concentration, the random effects of source herd and nursery room, and the repeated effect of time interval. Initial BW was used as a covariate for ADG in the repeated measures analysis. Effects of choline concentration on the discrete variables of fecal consistency score (1, 2, 3, or 4) and scouring frequency (number of days with fecal score ≥3 during each weekly period) were determined using a chi-squared test in PROC GLIMMIX. Linear and quadratic orthogonal contrasts of effects of choline concentration were tested for all responses and the heterogeneous autoregressive (ARH1) covariance structure was used in all analyses. Preplanned contrasts between individual treatment differences were also determined. For all analyses, data points with studentized residuals outside ±3.5 were removed as outliers. Statistical significance was declared at *P* ≤ 0.05 and trends acknowledged at 0.05 < *P* ≤ 0.10. Heteroscedasticity was determined by generating a histogram and normal quantile-quantile plot of residuals.

A linear decrease in the MR intake during d 1 to 14 was observed due to increased choline inclusion with C0 and C700 having greater MR consumption compared with C1400 and C2100 (*P* = 0.01). During d 15 to 28 MR intake was lowest in C1400, followed by C2100 (*P* < 0.01). From d 29 to 42 MR intake tended to be greatest for C700 followed by C2100 (*P* = 0.08) Finally, from d 1 to 49 the MR intake in C700 was greater compared with that of the other treatments (*P* < 0.01; Table 1). For CS intake on d 50 to 56, C700 had greater intake compared with that of C0 and C1400 (*P* = 0.01). When considering total feed intake by day for d 50 to 56, C700 had greater intake compared with C0 and C1400 (*P* = 0.01).

**Table 1:** Effect of increasing concentrations of choline in milk replacer on milk replacer and starter intake of dairy calves

Item	Treatment^1^				SEM	*P*-value^2^		
	C0	C700	C1400	C2100		Trt	Linear	Quad
Milk replacer intake, g/d								
d 1 to 14	584.3^a^	583.6^a^	568.6^b^	566.4^b^	2.21	0.04	0.01	0.95
d 15 to 28	655.0^a^	655.0^a^	652.1^c^	653.6^b^	4.29	<0.01	<0.01	<0.01
d 29 to 42	651.4^y^	655.7^x^	652.1^y^	653.6^xy^	29.0	0.11	0.08	0.20
d 43 to 49	163.6	163.6	162.9	163.6	0.05	0.99	0.99	0.99
d 1 to 49	583.7^b^	589.8^a^	581.6^b^	583.7^b^	2.24	<0.01	0.15	0.44
Calf starter intake, g/d								
d 1 to 14	9.89	15.1	18.8	22.4	4.35	0.18	0.03	0.83
d 15 to 28	141.7	171.8	179.2	175.8	24.7	0.71	0.33	0.50
d 29 to 42	398.8	531.1	429.8	508.4	54.1	0.27	0.36	0.62
d 43 to 49	546.3	670.4	575.8	635.0	45.1	0.21	0.40	0.47
d 50 to 56	986.4^b^	1,134^a^	940.9^c^	1,077^ab^	58.3	0.09	0.77	0.92
d 1 to 49	355.1	408.2	349.0	398.0	30.0	0.41	0.64	0.94
d 1 to 56	657.2	744.9	618.4	716.1	40.8	0.13	0.78	0.91
Total feed intake, g/d								
d 1 to 14	589.9	597.9	580.4	587.3	9.40	0.51	0.50	0.95
d 15 to 28	828.6	835.7	828.6	828.6	25.7	0.99	0.97	0.75
d 29 to 42	1,107	1,193	1,086	1,186	50.0	0.35	0.61	0.91
d 43 to 49	1,529	1,714	1,486	1,657	80.0	0.13	0.67	0.89
d 1 to 49	940.8	995.9	926.5	979.6	30.9	0.35	0.74	0.95
d 1 to 56	1,086	1,166	1,048	1,136	36.6	0.11	0.86	0.91

a–c Treatments with different superscripts within a row were considered different at *P* < 0.05.

x,y Treatments with different superscripts within a row exhibited a tendency for a difference (0.05 < *P* < 0.10).

1 Treatments included milk replacer with choline included at 0 (C0, n = 24), 700 (C700, n = 26), 1,400 (C1400, n = 25), or 2,100 (C2100, n = 25) mg/kg.

2 *P*-value of main effects of choline concentration (Trt) with linear and quadratic (Quad) contrasts.

Classical research suggests that dairy calves are born choline deficient and are reliant on choline from maternal colostrum to meet choline requirements (Waugh et al., 1947; Johnson et al., 1951). However, minimal research has focused on direct supplementation of pure choline to dairy calves on feed intake or performance of dairy calves. Molano et al. (2021) fed a blend of choline and B vitamins in calf starter from 4 to 13 wk of age. They observed no differences in DMI as a response to treatment. Recently, several studies have examined the impact of maternal rumen-protected choline (**RPC**) on growth and feed intake of their offspring. Swartz et al. (2022) observed no effect of feed intake on offspring of RPC-supplemented cows during the first 3 wk of life. Brown et al. (2023) observed that male Angus-Holstein calves had greater DMI when dams were fed RPC, but the effects appeared to be sex-specific because similar results were not observed in female calves. In mid-lactation adult Holstein cows, feeding unprotected choline at rates of 10 or 20 g/kg diet DM decreased DMI by approximately 8% (Sharma and Erdman, 1988), but similar results have not been observed in cows fed RPC (Sales et al., 2010). In the current study, the choline provided was not encapsulated. Therefore, the unprotected form of choline may have similarly decreased intake, especially when large amounts of MR were consumed during early life, perhaps due to decreased palatability.

During weaning, stress from the transition from liquid to solid feed can decrease feed intake of calves (Weary et al., 2008). In this study, it was observed that the calves that were supplemented with C700 of choline had greater CS intake after weaning compared with the other treatment groups. In lactating dairy cows, it has been observed that choline supplementation can increase postpartum DMI due to reduced inflammation and better lipid export from the liver (Humer et al., 2019). Our results indicate that choline supplementation may increase intake of MR and CS in calves as well, especially during the stressful weaning period. However, our results suggest providing choline above 2,350 mg/kg DM does not increase intake in calves.

The BW for calves in C700 tended to be greater compared with calves in C0 and C1400 (*P* = 0.10; Table 2). The difference in BW became more prominent at d 49 with the C700 group maintaining greatest BW (*P* = 0.01). On d 56, C700 had the greatest BW followed by C2100, and the lowest weight was the C1400 group (*P* < 0.01). Average daily gain differed among treatments in the preweaning period (*P* = 0.05). Specifically, during d 43 to 49, the ADG of C700 tended to be greater than that of the other treatments (*P* = 0.08). Likewise, over the entire 56-d trial period ADG was decreased in C0 and C1400 compared with C700 and C2100 (*P* < 0.01). Additionally, C2100 had greater d 56 hip heights compared with C0 and C1400 (*P* = 0.02).

**Table 2:** Effect of increasing concentrations of choline in milk replacer on growth of dairy calves

Item	Treatment^1^				SEM	*P*-value^2^		
	C0	C700	C1400	C2100		Trt	Linear	Quad
BW, kg								
d 1	38.9	40.3	39.4	39.7	0.91	0.51	0.64	0.38
d 14	43.1	44.7	42.7	43.8	0.74	0.16	0.98	0.73
d 28	49.9	52.0	49.8	51.3	0.95	0.17	0.65	0.73
d 42	58.5^y^	61.4^x^	58.6^y^	61.2^xy^	1.36	0.10	0.32	0.89
d 49	64.6^b^	69.2^a^	63.8^b^	67.1^ab^	1.60	0.01	0.72	0.61
d 56	72.5^b^	78.2^a^	69.0^c^	74.8^ab^	1.77	<0.01	0.78	0.96
ADG, kg/d								
d 1 to 14	0.29	0.29	0.30	0.29	0.03	0.99	0.94	0.93
d 15 to 28	0.49	0.54	0.50	0.53	0.03	0.38	0.45	0.64
d 29 to 42	0.66	0.72	0.64	0.72	0.03	0.18	0.55	0.83
d 43 to 49	0.86^y^	1.02^x^	0.88^y^	0.84^y^	0.06	0.08	0.44	0.08
d 50 to 56	1.11^b^	1.29^a^	0.91^c^	1.10^b^	0.07	<0.01	0.27	0.95
d 1 to 49	0.53^b^	0.59^a^	0.52^b^	0.56^ab^	0.02	0.05	0.76	0.65
d 1 to 56	0.62^b^	0.68^a^	0.55^b^	0.63^a^	0.02	<0.01	0.38	0.65
HH,^3^ cm								
d 1	81.9	81.7	81.1	82.4	0.66	0.41	0.75	0.20
d 56	92.7^b^	93.6^ab^	92.1^b^	94.5^a^	0.56	0.02	0.15	0.19
HH gain	10.7	11.9	10.9	11.7	0.72	0.22	0.35	0.62

a–c Treatments with different superscripts within a row were considered different at *P* < 0.05.

x,y Treatments with different superscripts within a row exhibited a tendency for a difference (0.05 < *P* < 0.10).

1 Treatments included milk replacer with choline included at 0 (C0, n = 24), 700 (C700, n = 26), 1,400 (C1400, n = 25), or 2,100 (C2100, n = 25) mg/kg.

2 *P*-value of main effects of choline concentration (Trt) with linear and quadratic (Quad) contrasts.

3 HH = hip height.

Calves in the C700 group had greater ADG and greater hip height compared with the C0 supplementation treatment group (*P* < 0.05). Additionally, C700 had improved gains and maintained increased intake compared with the C1400 calves. Including basal choline concentrations in MR, calves supplemented with 700 mg/kg received a total of 2,350 mg/kg DMb which is 1,350 mg/kg DM greater than the recommended 1,000 mg/kg DM. Although our results suggested that some choline supplementation helped in calf growth, Molano et al. (2021) did not observe any growth differences between calves fed a B vitamin and choline blend compared with calves that did not receive the blend during the transition and postweaning phase. Discrepancy in results could have been due to the time in which supplementation began. In the current study supplementation began within the first few days of life, whereas Molano et al. (2021) supplemented beginning at 4 wk. Likewise, even though the calves in the current study were fed for a longer duration before weaning compared with Molano et al. (2021), differences in growth and ADG were not observed until wk 6 or 7 of treatment implementation. Thus, there may be a delay associated with observed differences.

The C2100 tended to decrease feed efficiency by 12.1%, 10.5%, and 13.5% during d 43 to 49 compared with C0, C700, and C1400, respectively (*P* = 0.01; Table 3). However, from d 50 to 56, the C1400 had a 11.5% and 14.8% decreased G:F compared with C0 and C700 (*P* < 0.01). The C700 treatment group had greater G:F on d 50 to 56, corresponding to the greatest ADG for that time frame compared with the calves in the other treatments. In Sharma and Erdman (1988), cows supplemented with greater choline concentrations not only had reduced feed intake but also had decreased milk production. In the current study greater supplementation of choline did not improve feed efficiency or growth in the calves assigned to that treatment. To the authors' knowledge, no studies have considered adverse effects of choline supplementation in MR on intake and growth of dairy cows; however, it seems that oversupplementation does not improve feed efficiency. Overall, even though C700 consumed more MR and CS during this period, they were more efficient than the calves in the other groups as they gained more kilograms per kilogram of feed than they consumed.

**Table 3:** Effect of increasing concentrations of choline in milk replacer on feed efficiency of dairy calves

G:F, kg/kg	Treatment^1^				SEM	*P*-value^2^		
	C0	C700	C1400	C2100		Trt	Linear	Quad
d 1 to 14	0.50	0.45	0.50	0.49	0.04	0.69	0.90	0.52
d 15 to 28	0.61	0.64	0.60	0.64	0.04	0.39	0.68	0.78
d 29 to 42	0.60	0.58	0.58	0.61	0.03	0.38	0.53	0.12
d 43 to 49	0.58^x^	0.57^x^	0.59^x^	0.51^y^	0.02	0.08	0.09	0.14
d 50 to 56	0.52^x^	0.54^x^	0.46^y^	0.49^xy^	0.01	0.07	0.13	0.87

x,y Treatments with different superscripts within a row exhibited a tendency for a difference (0.05 < *P* < 0.10).

1 Treatments included milk replacer with choline included at 0 (C0, n = 24), 700 (C700, n = 26), 1,400 (C1400, n = 25), or 2,100 (C2100, n = 25) mg/kg.

2 *P*-value of main effects of choline concentration (Trt) with linear and quadratic (Quad) contrasts.

Average fecal score did not differ among treatments throughout the entire preweaning period (C0: 1.34, C700: 1.41, C1400: 1.40; C2100: 1.37; χ^2^ = 2.83; *P* = 0.42) or during the postweaning period (C0: 1.02, C700: 1.04, C1400: 1.00; C2100: 1.03; χ^2^ = 3.02; *P* = 0.39). Furthermore, no difference was observed during the entire 56-d experiment (C0: 1.30, C700: 1.37, C1400: 1.35; C2100: 1.33; χ^2^ = 2.52; *P* = 0.47). Scouring frequencies also did not differ among the groups during any week of the experiment. Our results agree with those by Swartz et al. (2022), who observed no differences in risk of neonatal calf diarrhea in calves born from dams that were supplemented with different levels of RPC.

Due to high choline concentrations within basal MR, all treatments provided greater daily choline supply (1,650 mg/kg DM) than current NASEM (2021) recommendations of 1,000 mg/kg DM. Nevertheless, even with these high basal concentrations, additional benefits to MR intake and ADG were observed with further supplementation of 700 mg/kg (2,350 mg/kg DM). These effects were particularly strong immediately postweaning. No additional increases in feed intake or gain were observed at choline concentrations above 2,350 mg/kg. However, it is important to note that the calves in the current study were fed concentrations of MR that are lower than the current NASEM recommendations (>680 g/d) and in the current study conditions 2,350 mg/kg supplementation appeared to be closer to the optimal requirement for growth. Future research should continue to revisit choline requirements for young dairy calves.
